# Preaxial polydactyly of the foot in an adult patient diagnosed by X-ray after a trauma

**DOI:** 10.1016/j.radcr.2022.12.034

**Published:** 2023-01-24

**Authors:** Anna Russo, Vittorio Patanè, Vittorio Viglione, Alessandro Pinto, Mariarita Cristiano, Rosita Comune, Luigi Gallo, Alfonso Reginelli

**Affiliations:** Department of Precision Medicine, University of Campania Luigi Vanvitelli, Naples, Italy

**Keywords:** Polydactyly, Preaxial, Foot polydactyly, Adult polydactyly, X-ray

## Abstract

Polydactyly, or hyperdactyly, is a frequent malformation, with a reported incidence between 0.37 and 1.2 per 1000 live births. Most cases encountered in medical practice are sporadic cases, usually presenting one-sided manifestations. More frequently, polydactyly can be detected prenatally through an ultrasound examination, if not, it is usually diagnosed at birth, during the first physical examination. Although the clinical diagnosis is relatively easy in patients with manifest cases, it can sometimes be present with mild or partial forms that are not clinically evident on physical examination, resulting in later diagnosis and treatment. We reported a particular case of polydactyly of the foot not clinically manifest, diagnosed in a 39-year-old Caucasian Male patient with a history of recurrent localized pain in the big toe often associated with subungual bleeding, since he was a child who came to our emergency room following a car accident. Polydactyly is a frequently reported congenital malformation which may present in many different varieties of deformities. In this case, the X-ray, which was required after a car accident, leads to the incidental diagnosis of polydactyly in an adult patient. As described, because of the functional limitations related to this malformation, as well as to limit recurrent pain, and subungual anomalies, the patient underwent to a surgical correction to improve its quality of life.

## Background

Polydactyly, also known as hyperdactyly, is a frequently reported malformation, with a reported incidence between 0.37 and 1.2 per 1000 live births [1,2]. Both hand and foot polydactyly may present various morphological phenotypes, which have been correlated with different functional and aesthetic implications. Consequently, to prevent long-term complications, this condition often requires an early surgical resolution [2]. Polydactyly has been found to be linked to a defect during the early stages of limb development, which may be associated with genetic or teratogenic factors interfering with the molecular mechanism of fingers development [3,4]. Morphogenetically, polydactyly may be considered as a process of bifurcation of the rays of the fingers in the longitudinal axis, progressing from the distal to the proximal end [3]. The bifurcation process occurs in numerous degrees and consequently the deformity appears in many varieties and severity, ranging from a widening of a distal phalanx to a complete duplication of one or more toe radii with involvement of the carpal or tarsal bones [5]. Familiarity has sometimes been reported, particularly, when polydactyly presents as the only deficit, it is most commonly linked to an autosomal dominant inheritance pattern. However, most cases encountered in medical practice are sporadic cases, usually presenting one-sided manifestations [6,7]. More frequently, polydactyly can be detected prenatally through an ultrasound examination, if not, it is usually diagnosed at birth, during the first physical examination [6]. Once diagnosed, radiographic investigations must be performed to evaluate and define the supernumerary bony elements present. Although the clinical diagnosis is relatively easy in patients with manifest cases, it can sometimes be present with mild or partial forms that are not clinically evident on physical examination, resulting in later diagnosis and treatment. In these cases, the X-ray represented the diagnostic investigation of choice that found these anomalies, resulting also useful for planning any surgery. Herein, we report a particular case of polydactyly of the foot not clinically manifest, diagnosed at the age of 39 in a patient with an history of chronic foot pain, and in which a diagnosis of polydactyly was made following an X-ray taken after trauma.

## Case report

**Fig. 1:**
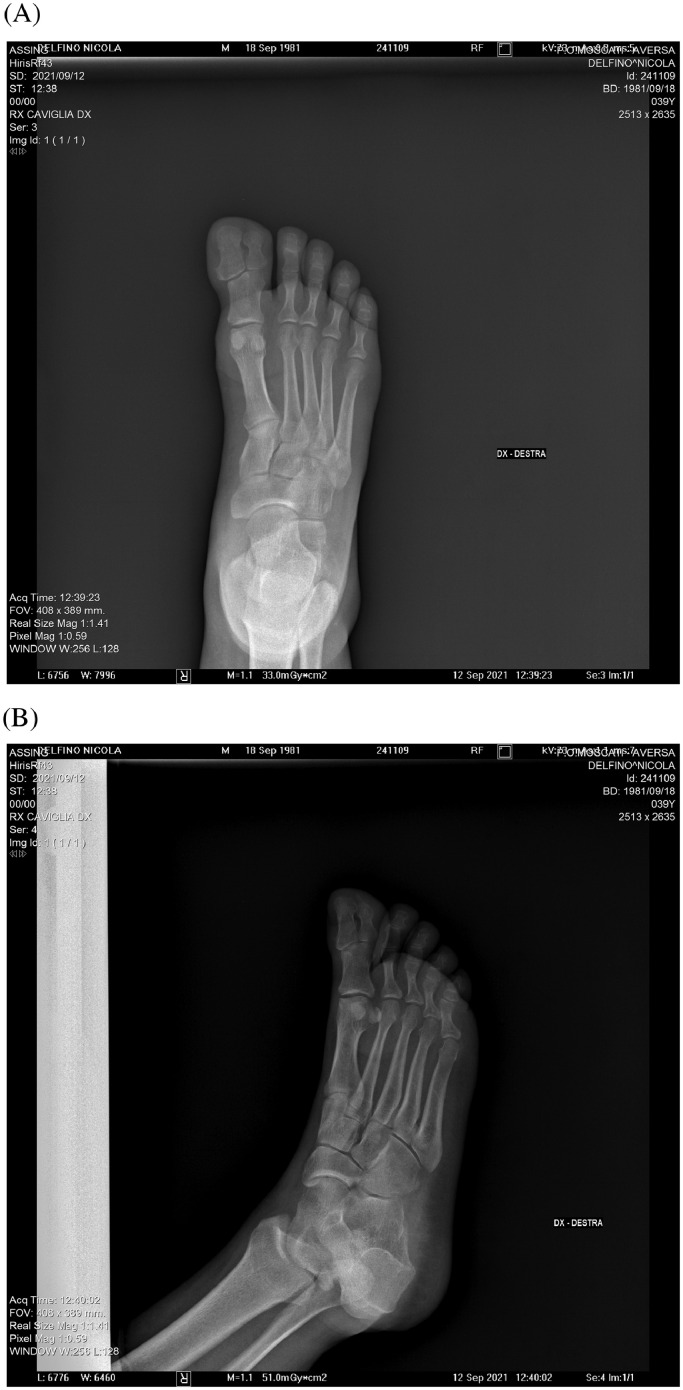
Anterior posterior (A) and laterolateral (B) radiograph of the right foot showing the distal phalangeal type of preaxial polydactyly.

A 39-year-old Caucasian male came to our emergency room referring a severe pain of his right foot and lower limb following a car accident. His medical history was negative, except for hypertension. X-rays were therefore required to search for any fractures of the limb and foot. The radiographic examination did not show any fractures; however, the radiological images of the foot revealed the presence of 2 distal phalanges of the right big toe, being compatible with the diagnosis of preaxial polydactyly of the right foot (Fig. 1). The contralateral foot was examined too for a comparative evaluation, and any anomalies were depicted. Interestingly, the patient denied of being aware of this congenital malformation or a familiar recurrence history of known polydactyly or any other malformation; however, he reported a long-lasting history of recurrent localized pain in the big toe often associated with subungual bleeding, since he was a child, for which he had never made any diagnostic investigations. No bone fracture was found on the X-ray, but the evidence of the distal supernumerary phalanx resulted diagnostic for polydactyly. Hence, due to the functional limitations related to this malformation, as well as to limit recurrent pain and subungual anomalies reported by the patient, surgical correction was required. Unfortunately this case has happened in a traumatologic orthopedic center and for this reason, patient has been sent to a surgical orthopedic center in which underwent a surgical removal of the supernumerary bony skeleton. Given that surgical treatment was performed in another hospital, no further information has been recovered.

## Discussion

Polydactyly is a frequently reported congenital malformation which may present in many different varieties of deformities, ranging from single extra digits to complex central foot duplications with tarsal or carpal bone duplication [1,2]. From a topographical point of view, polydactyly may be classified in polydactyly of the hand and foot. Foot polydactyly can be distinguished as preaxial (affecting the big toe), postaxial (more common, affecting the fifth toe), and central (rarer, affecting one or more of the 3 central toes) [8]. Despite the high prevalence of polydactyly of the hand and foot in infants, preaxial of the foot is rare. Indeed, the duplication of the big toe is rarer than the polydactyly of the thumb, with reported incidence rates of 0.024/1000 and 0.165/1000 respectively [6,8]. The duplication of the big toe has been reported more frequently in males, mainly affecting the right foot, and being more frequently one-sided (81.5%) [9,10]. Even if the exact pathophysiology of isolated polydactyly remains unknown, a possible correlation with maternal diabetes has been suggested, but data from literature are still poor [11]. Although genetic transmission (autosomal dominant pattern) has sometimes been described, particularly when polydactyly presents as the only deficit, most reported cases are sporadic cases, usually presenting as one-sided manifestation [[3], [4], [5]]. On the other side, familial types are mostly bilateral and symmetrical, while the isolated presentation is more frequent than the syndromic aspect [7,8]. Preaxial polydactyly of the foot can be associated with complex syndromic pictures including more severe malformations, such as craniosynostosis, syndactyly, atrial septal defects, and agenesis of the corpus callosum [12]. Hence, it is important to know the various clinical presentations of preaxial polydactyly in order to reach a faster diagnosis also for these linked severe syndromes and malformations. However, due to the low prevalence, most clinicians are unfamiliar with these congenital malformations and associated syndromes, which may therefore not be recognized at an early stage. Furthermore, the multiple phenotypes of polydactyly can range from a skin protuberance smaller than a finger to the doubling of a single phalanx [13]. The variability of clinical presentations, especially for less manifest cases may delay the diagnosis and consequently the surgical treatment. As regard diagnosis, ultrasound may be used to early detect these malformations during pregnancy. When prenatal diagnosis is not reached, the first physical examination at the time of birth usually leads to detect and diagnosis manifest cases of polydactyly [14]. In case of polydactyly diagnosis, an X-ray examination should be performed to determine whether any bone elements are present in the additional digit [9]. X-ray may also result useful to check for eventual underlying syndrome or any additional skeletal abnormalities. Furthermore, radiographic examination is required to choose the best surgical approach to remove the additional digit [17,18]. Even if the diagnosis is frequently reached prenatally by ultrasound examination, or at the time of birth, by the first physical examination, some cases may present as fewer manifest malformations, which may not be appreciated in a routing physical examination. Hence, the diagnosis may be a challenge for physicians in these less manifest cases. However, it is important to underline the need to make an early diagnosis even in these cases, in order to exclude other possible congenital anomalies and associated syndromes, which may require an early specific treatment [13,14,19]. Although rare, some cases have been described in the literature in which minimal alterations (not visible on neonatal physical examination), were instead diagnosed in more advanced stages, due to subsequent symptoms reported by patients, or more often by parents, with a variable impact on patients’ quality of life, such as the need for these patients to use 2 different shoe sizes or duplicated toe pain, or recurrent nail bleeding from a toenail [15,16]. Hiraoka et al. [15] reported the case of a healthy 2-year-old girl which referred their center due repeated episodes of occasional bleeding from the right toenail. Physical examination revealed a normal looked toe, except for the second nail on her right foot, which was wider from the third to the fifth and was separated in the center. In this case, an X-ray examination revealed a duplicated second distal phalanx, and was crucial for choosing the best surgical approach [15]. We reported a particular case of polydactyly of the foot not clinically manifest, diagnosed in 39 years old, a patient with a history of recurrent localized pain in the big toe often associated with subungual bleeding, since he was a child. In this case, the X-ray, which was required after a car accident, leads to the incidental diagnosis of polydactyly in an adult patient. As described, because of the functional limitations related to this malformation, as well as to limit recurrent pain, and subungual anomalies, the patient underwent to a surgical correction to improve its quality of life.

## Conclusion

Preaxial polydactyly is therefore an infrequent condition that is generally easily diagnosed in the classic clinically manifest forms, but which can remain undiagnosed for a long time in the forms that are not clinically evident. A key role is associated with the use of radiology to evaluate and better define the different types of bone anomalies in the limbs, and any other related anomalies. In addition, radiology is decisive in the indication in the choice of the surgical correction to be carried out to eliminate the supernumerary bone portion. The use of X-ray in polydactyly is therefore essential both for the diagnosis but also for the surgical and therapeutic approach to be carried out.

## Patient consent

Informed written consent was obtained from the patient for publication of the case report and all imaging studies. Consent form on record.
